# Developing the Additive Systems of Stand Basal Area Model for Broad-Leaved Mixed Forests

**DOI:** 10.3390/plants13131758

**Published:** 2024-06-25

**Authors:** Xijuan Zeng, Dongzhi Wang, Dongyan Zhang, Wei Lu, Yongning Li, Qiang Liu

**Affiliations:** 1 College of Forestry, Hebei Agricultural University, Baoding 071001, China; zengxj7@126.com (X.Z.); sanpangzi1228@126.com (W.L.); yongninghao@163.com (Y.L.); qiangliu2015@126.com (Q.L.); 2 College of Economics and Management, Hebei Agricultural University, Baoding 071001, China; zhdys@163.com

**Keywords:** stand basal area model, adjustment in proportion, nonlinear seemingly unrelated regression, *Populus davidiana*, *Betula platyphylla*, broad-leaved mixed forests

## Abstract

Stand basal area (SBA) is an important variable in the prediction of forest growth and harvest yield. However, achieving the additivity of SBA models for multiple tree species in the complex structure of broad-leaved mixed forests is an urgent scientific issue in the study of accurately predicting the SBA of mixed forests. This study used data from 58 sample plots (30 m × 30 m) for *Populus davidiana* × *Betula platyphylla* broad-leaved mixed forests to construct the SBA basic model based on nonlinear least squares regression (NLS). Adjustment in proportion (AP) and nonlinear seemingly unrelated regression (NSUR) were used to construct a multi-species additive basal area prediction model. The results identified the Richards model (M_6_) and Korf model (M_1_) as optimal for predicting the SBA of *P. davidiana* and *B. platyphylla*, respectively. The SBA models incorporate site quality, stand density index, and age at 1.3 m above ground level, which improves the prediction accuracy of basal area. Compared to AP, NSUR is an effective method for addressing the additivity of basal area in multi-species mixed forests. The results of this study can provide a scientific basis for optimizing stand structure and accurately predicting SBA in multi-species mixed forests.

## 1. Introduction

Stand basal area (SBA) is a crucial indicator for evaluating site quality [1], predicting stand productivity [2], and optimizing stand structure [3], as well as an important variable in models for predicting stand volume [4], biomass [5,6], and carbon storage [7,8]. Recent studies on SBA have mainly focused on plantations with simple stand structures [9,10]. In contrast, investigations into the SBA of structurally complex multi-species forests remain relatively scarce [11]. Therefore, exploring the dynamics of SBA in mixed stands holds significant implications for optimizing stand structure [12,13], refining management practices [14,15] and mitigating the impacts of climate change [16].

Models for predicting basal area are important for understanding forest tree diameter at breast height (DBH) [17] as well as basal area growth and development patterns [18]. Basal area models exist at various levels, ranging from individual tree [19,20] to stand-level [21,22] models. Linear models [23,24], nonlinear models [25,26], and logarithmic transformation linear models [27,28] are based on the characteristics of different stand types and tree species and are commonly used to study variations in basal area. Linear models [24] and logarithmic transformation linear models [29] are often utilized to characterize the growth patterns of basal area in individual trees. In contrast, SBA dynamics are typically investigated using nonlinear models [30]. Some studies [31,32] have identified nonlinear models to provide an improved representation of the biological characteristics of forest trees when predicting SBA, resulting in predictions of higher accuracy and applicability. The reliability of a stand basal area model in predicting the SBA of complex, structured mixed forests directly affects forest management decisions [33]. Consequently, the construction of a model for predicting the SBA of mixed forests is of considerable significance for optimizing forest management measures [34].

The predictive accuracy of SBA models is typically influenced by a diverse range of factors and their combinations, including site quality [35,36], stand age [23,37], stand density [38,39], and tree species composition [40,41]. Site quality is an important variable for constructing mixed forest SBA models and is positively correlated with SBA [16,36]. However, this positive relationship between site quality and basal area varies among different tree species in a mixed forest [11]. Furthermore, stand density is the main indicator of competition between tree species [42] and among trees [20]. Liu et al. (2022) [43] constructed a broad-leaved mixed forest SBA prediction model that included stand density variables. The accuracy with which models can predict mixed forest SBA is also dependent on the biological characteristics of mixed forests [37,44], interactions among tree species [19], and forest spatial distribution patterns [45]. The main factors influencing SBA differ among different forest types [37,44,46]. Therefore, exploring the key factors influencing model predictions of mixed forest SBA is of significance for optimizing forest structure.

Model accuracy in predicting SBA is influenced by numerous biotic [23] and abiotic [36] factors, as well as by the different parameter estimation methods, including nonlinear least squares (NLS) [30,47], nonlinear mixed-effects model (NLME) [48], adjustment in proportion (AP) [49], and nonlinear seemingly unrelated regression (NSUR) [50]. NLS is sensitive to data and can only be applied under specific conditions [51,52], including the assumption of constant variance and independent errors [30,53]; NLME has various drawbacks, including a requirement for numerous model parameters and a tendency towards non-convergence [54]. However, the SBA of multi-species mixed forests is influenced by the tree species in that forest [37,55]. AP and NSUR have been widely applied in additive models predicting SBA [49,50]. AP directly categorizes SBA among tree species in a mixed forest according to a weighting approach [56]. The AP approach has been shown to be more accurate for expressing SBA among different tree species in a mixed forest and can ensure that the summed basal area aligns with total SBA [57]. However, AP cannot explain the inherent correlation between tree species [58], whereas NSUR can effectively overcome this challenge [59]. Given the improved flexibility and versatility of NSUR compared to AP [60]. Past studies have explored different parameter estimation methods for application to models for predicting mixed forest SBA [30]. However, the impact of different additive parameter estimation methods on model accuracy is uncertain. Therefore, choosing the appropriate additive parameter estimation method is important for accurately predicting the SBA of broad-leaved mixed forests.

*Populus davidiana* × *Betula platyphylla* broad-leaved mixed forests are a major forest type in China, playing a significant role in improvements in forest structure [13,43], enhancement of forest productivity [61], and climate regulation [62]. Some previous studies [50,63] have shown that various factors and parameter estimation methods influence SBA. However, in multi-species broadleaf mixed forests, exploring the additivity of basal area models for different tree species is a current key issue in accurately predicting the stand basal area. Therefore, the present study used *P. davidiana* × *B. platyphylla* broad-leaved mixed forests in the Mulan Rangeland State Forest of Hebei Province as the research object. The objectives of the present study were to: (1) identify the optimal basic model for predicting the SBA of *P. davidiana* and *B. platyphylla* based on NLS; (2) improve the above model by separately using AP and NSUR while ensuring the additivity of SBA prediction in broad-leaved mixed forests; and (3) assess the relative improvements in model accuracy by estimating model parameters using a diverse range of parameter estimation techniques. The results of the present study can act as a scientific foundation for optimizing stand structure, improving stand productivity, and improving mixed forest management measures.

## 2. Results

### 2.1. Basic Model for Predicting the SBA of Populus davidiana × Betula platyphylla Broad-Leaved Mixed Forests

As shown in Table 1 and Table 2, the Richards model (M_6_), which incorporates site quality (SI), stand density index (SDI), and age at 1.3 m above ground level (ADBH), was optimal for describing the SBA of *P. davidiana*, achieving $R_{adj}^{2}$, MAE, MPE, and RMSE statistics of 0.962, 0.973, 0.070, and 1.438, respectively. The Korf model (M_1_), which incorporates SI, SDI, and ADBH, was optimal for predicting the SBA of *B. platyphylla*, achieving $R_{adj}^{2}$, MAE, MPE, and RMSE statistics of 0.961, 0.515, 0.091, and 0.749, respectively.

### 2.2. Nonlinear SBA Models Fitted Using AP

As shown in Table 3, a model for predicting the SBA of *P. davidiana* × *B. platyphylla* broad-leaved mixed forests was fitted and evaluated using AP based on the optimal theoretical model identified in Table 2. Since SI and SDI showed positive correlations with the SBA of *P. davidiana* and *B. platyphylla*, respectively, their addition contributed to increased model predictive precision. The $R_{adj}^{2}$ and RMSE of the *P. davidiana* SBA theoretical model were 0.981 and 0.709, respectively; the $R_{adj}^{2}$ and RMSE of the *B. platyphylla* SBA theoretical model were 0.979 and 0.678, respectively; and the $R_{adj}^{2}$ and RMSE of the total SBA theoretical model were 0.939 and 1.776, respectively.

### 2.3. Nonlinear SBA Models Fitted Using NSUR

As shown in Table 4, a model for predicting SBA of *P. davidiana* × *B. platyphylla* broad-leaved mixed forests was fitted and evaluated using NSUR, based on the optimal theoretical model identified in Table 2. The incorporation of NSUR increased the prediction accuracy of SBA at both the tree species level and the stand level. After inspection and evaluation, the $R_{adj}^{2}$ and RMSE of the model for predicting SBA of *P. davidiana* were 0.9870 and 0.6297, respectively; the $R_{adj}^{2}$ and RMSE of the model for predicting SBA of *B. platyphylla* were 0.9858 and 0.4092, respectively; the $R_{adj}^{2}$ and RMSE of the model for predicting the total SBA were 0.9757 and 0.6263, respectively.

### 2.4. Model Evaluation and Prediction

The present study evaluated the models for predicting the tree species- and stand-level basal area within *P. davidiana* × *B. platyphylla* broad-leaved mixed forests using MAE, MPE, RMSE, R^2^, and $R_{adj}^{2}$ (Table 5). The additive model for predicting SBA of *P. davidiana* × *B. platyphylla* broad-leaved mixed forests was constructed based on the NSUR approach, which showed higher prediction accuracy and applicability at both the tree species and stand level in comparison to the NLS and AP approaches. Compared to NLS, the R^2^ of the model using NAP to describe SBA_t_ improved by 2.24%, while the MAE, MPE, and RMSE decreased by 31.98%, 31.88%, and 34.12%, respectively. Similarly, compared to NLS, the R^2^ of the model using NSUR to describe SBA_t_ increased by 3.94%, and the MAE, MPE, and RMSE declined by 64.48%, 36.23%, and 64.75%, respectively.

**Figure 1:**
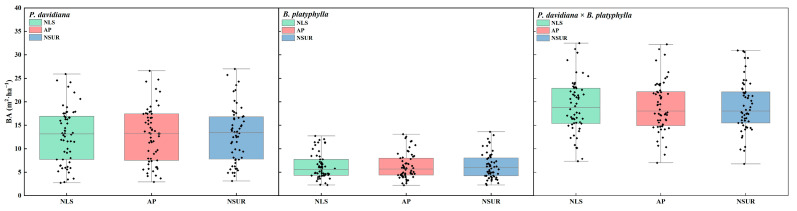
Prediction of stand basal area in *Populus davidiana* × *Betula platyphylla* broad-leaved mixed forests based on different parameter estimation methods.

**Figure 2:**
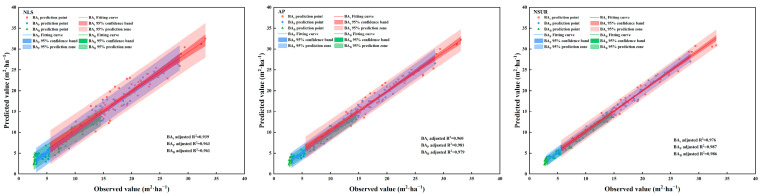
Comparison of model predicted and observed stand basal area for *Populus davidiana* × *Betula platyphylla* broad-leaved mixed forests.

NLS, AP, and NSUR were adopted based on the identified optimal theoretical model for SBA of *P. davidiana* (M_6_) and *B. platyphylla* (M_1_) to simulate the SBA in mixed broadleaved forests (Figure 1), and the effects of different parameter estimation methods on model prediction accuracy were compared. Figure 2 shows the variance between predicted and observed values among the approaches. The results showed that the model based on NSUR achieved higher accuracies of simulated SBA compared to the models based on NLS and AP, with $R_{adj}^{2}$ for the total SBA model, *P. davidiana* SBA model, and *B. platyphylla* SBA model of 0.976, 0.987, and 0.986, respectively. The model showed good consistency between predicted and observed values of SBA at both tree species and stand levels.

## 3. Discussion

The present study examined six forms of theoretical models for predicting SBA based on the Korf, Schumacher, and Richards models. The application of NLS showed that the Richards model (M_6_) and Korf model (M_1_) were optimal for describing the SBA of *P. davidiana* and *B. platyphylla*, respectively. An additive model for predicting the SBA of *P. davidiana* × *B. platyphylla* broad-leaved mixed forests was then constructed using AP and NSUR. In comparison to the model using AP, the model using NSUR showed a higher prediction accuracy. Therefore, the model for predicting the SBA of *P. davidiana* × *B. platyphylla* broad-leaved mixed forests using NSUR can provide a scientific basis for improving the structure of mixed forests, increasing productivity, and optimizing forest management.

### 3.1. Selection of an Optimal Model for Predicting SBA

When applied to the *P. davidiana* × *B. platyphylla* broad-leaved mixed forests, the Richards model (M_6_) and the Korf model (M_1_) were optimal for predicting the SBA of *P. davidiana* and *B. platyphylla*, respectively (Table 2). These results show that optimal models for predicting tree species-level basal area differ among different species due to differences in biological characteristics [65]. When compared to the Schumacher model, the Richards model exhibited improved mathematical properties and biological significance [33]. Consequently, the Richards model has seen wide application for predicting the SBA of plantations [10,66] and mixed stands [2,67], consistent with the model selected by Fu et al. (2017) [2] for predicting the SBA of broad-leaved mixed forests dominated by Mongolian oak. A comparison of the Korf model with the Richards and Schumacher models in the present study showed that the former displayed improved performance in predicting the SBA of *B. platyphylla* [68]. Within mixed forests, biological characteristics [69], interspecific effects [44], and spatial distribution patterns [37] of different tree species affect the tree growth and development process [70]. Hence, there is a greater emphasis on selecting the model for predicting SBA that optimizes prediction accuracy and model applicability.

### 3.2. Factors Affecting the SBA Model

SI, SDI, and ADBH have been shown to be the main factors affecting models for predicting SBA [33,50]. The present study observed a positive correlation between ADBH and SBA within the *P. davidiana* × *B. platyphylla* broad-leaved mixed forests at both the tree species level and stand level (Table 4), consistent with the findings of Poage and Tappeiner (2002) [71] for Douglas fir. In contrast, Smith and Long (2001) [72] identified a negative correlation between stand age and SBA. Taylor et al. (2020) [44] concluded that stand age is an important factor influencing both tree size and its diameter distribution patterns [73,74], and that inter-tree competition intensifies with increased stand age [75,76].

Both the tree species-level and stand-level basal areas showed a positive correlation with SI in the *P. davidiana* × *B. platyphylla* broad-leaved mixed forest (Table 4). This result was consistent with simulations of SBA for Norway spruce by Yue et al. (2012) [63]. Donoso and Soto (2016) [36] confirmed that the improved quality of a site can result in increased SBA. In contrast, Padilla-Martínez et al. (2024) [77] claimed that the basal area of some tree species was negatively correlated with site quality. These contrasting results can possibly be attributed to the structure of the canopy and the efficiency of photosynthesis [16]. Condés et al. (2013) [78] showed that while the accuracy of SBA prediction models depends on site quality, there is also a correlation with the stand density.

An increase in SDI will gradually increase the tree species-level and stand-level basal area in the *P. davidiana* × *B. platyphylla* broad-leaved mixed forests (Table 4), eventually stabilizing the SBA at a higher density [76]. The number of trees per hectare and SDI are common indicators reflecting stand density in different forest types [43,79], with the latter extensively employed in the construction of a forest growth and harvest model [80,81]. The results of the present study showed that the incorporation of SDI achieved increased accuracy in predicting tree species-level and stand-level basal area (Table 1). He et al. (2021) [50] similarly introduced SDI into an SBA prediction model when studying the SBA of natural oak forests. SDI has been shown to be important for exploring changes in SBA [43]. While Ruiz-Benito et al. (2014) [3] proposed that climate factors were the main factors affecting the SBA, these effects differ among different tree species [28]. Since the present study did not investigate the influence of climatic variables on SBA, future studies should concentrate on examining the impact of climatic factors on the SBA model.

### 3.3. Parameter Estimation Methods Affect the Accuracy of SBA

The model incorporating NSUR achieved higher accuracy for predicting the SBA of *P. davidiana* × *B. platyphylla* broad-leaved mixed forests compared to models incorporating NLS and AP (Table 5 and Figure 2). This result is consistent with the results of the crown prediction model constructed by Fu et al. (2017) [58]. Past studies have similarly demonstrated that the choice of parameter estimation method influences the accuracy of the SBA model [82,83]. While the NLS is most widely used in the construction of SBA simulation models [84,85], this approach requires the model error to meet the assumptions of an independent normal distribution, an absence of autocorrelation, and non-collinearity [86]. Consequently, the method is not applicable to non-normally distributed time-series SBA data [10]. In addition, NLS cannot guarantee that summing the SBA of each tree species will equate to the total SBA [58]. Therefore, the selection of an appropriate parameter estimation method for the development of an additive model to predict the SBA of a mixed forest is crucial.

Compared with AP, NSUR effectively solves the problem of the additivity of the SBA of tree species in mixed forests (Table 5 and Figure 2). Past studies have similarly demonstrated that NSUR is effective in solving the additivity problem and has higher prediction accuracy [56,57]. Using AP, errors in model prediction [57] of the SBA of mixed forest can be attributed to the lack of consideration of the inherent correlations between the SBAs of different tree species [87], consistent with the conclusions of Fu et al. (2017) [58] and Lei et al. (2018) [57]. The incorporation of NSUR effectively overcomes the above challenge [58,59], thereby allowing unbiased estimation of model parameters [58] and improving the efficiency of parameter estimation [83]. Therefore, the NSUR has been shown to be effective for the development of an additive SBA prediction model for different tree species in mixed forests.

Some past studies [88,89,90] have shown that sample plot size and sample quantity may affect the accuracy of model predictions of the SBA of mixed forests. The cross-validation technique can be used to limit errors when using limited sample size data [91], and ten-fold cross-validation is often used for the SBA model evaluation [28,92]. Since the present study was based on data from 58 sample plots, the accuracy of the constructed SBA prediction model was optimized by employing the ten-fold cross-validation method. In addition, some previous studies [90,93] have asserted that sample plot size may affect the prediction accuracy and applicability of mixed forest SBA models. He et al. (2021) [50] and Padilla-Martínez et al. (2024) [77] constructed SBA models for natural oak forests and Mexican temperate multi-tree forests using sample plots of 0.06 ha and 0.25 ha, respectively. The present study constructed a high-precision SBA prediction model for broad-leaved mixed forests using a sample plot area of 0.09 ha (30 m × 30 m), consistent with the sample plot area used by Monserud and Sterba. (1996) [94] when constructing an SBA prediction model for Austrian Norway spruce and Scots pine mixed forests. Future studies should further examine the influence of sample plot area and sample quantity on the predictive accuracy and practicality of mixed-stand SBA models.

## 4. Materials and Methods

### 4.1. Study Area

The study area of the present study is in Mulan Rangeland State Forest, Hebei Province, China (41°35′–42°40′ N, 116°32′–117°14′ E), and is forest with an area of 9.05 × 10^4^ ha, forest cover of 85%, total forest volume of 5.56 × 10^6^ m^3^, and an altitude of 750–1998 m. The study area falls into a continental monsoon and mountainous climate zone [93] with an average annual precipitation of 380–560 mm, an average annual temperature of −1.4–4.7 °C, and a frost-free period of 67–128 d. The range of primary tree species found in the study area includes *P. davidiana*, *B. platyphylla*, *Larix principis-rupprechtii*, *Picea asperata*, and *Quercus mongolica*; the principal shrub species comprise *Corylus mandshurica*, *Hippophae rhamnoides*, and *Rhododendron micranthum*; and the main herbs include *Carex tristachya*, *Thalictrum aquilegifolium*, and *Polygonatum odoratum*.

### 4.2. Data Description

The present study sourced data from 58 sample plots (30 m × 30 m each) [95] from the study area between 2013 and 2023. Information for standing trees with a DBH ≥ 5 cm in each sample plot was recorded, including count, relative coordinates, height, crown width, DBH, and other tree factors. Site factors in each sample plot, including elevation, gradient, and slope, as well as stand factors, including average DBH, density, average height, and mixing ratio, were also recorded. Five average trees for each species were selected in each sample plot. The growth cone [96] method was used to extract tree cores at breast height, which was used to determine ADBH [97]. Five dominant trees [50] of each species in each plot were also selected, and their heights were measured, with the mean calculated to determine the height of individual tree species and the entire stand. The sample plots fell within an elevation range of 1233 m to 1802 m and a slope range of 0° to 23°. A statistical summary of stand-level and tree species-level data is provided in Table 6.

### 4.3. Methods

#### 4.3.1. Basic Model Selection

The SBA model can be expressed both theoretically and empirically [98], with the former being highly logical and containing parameters with biological significance [38]. Recent studies [2,21,30] have constructed individual tree-level [65] and stand-level [33,38] basal area prediction models for various stand types based on theoretical models developed by Korf, Schumacher, and Richards. Therefore, the present study adopted different forms of theoretical models (Table 7) to construct an SBA prediction model for a *P. davidiana* × *B. platyphylla* mixed broadleaved forest.

#### 4.3.2. Adjustment in Proportion (AP)

The model must ensure that the sum of the SBA for each species equals the total SBA of the *P. davidiana* × *B. platyphylla* broad-leaved mixed forests [101]. AP uses a weighting approach for tree species SBA [57] to predict both the SBA of each species as well as the total SBA of the mixed forest [58]. The present study constructed an AP-based SBA prediction model for *P. davidiana* × *B. platyphylla* broad-leaved mixed forests (Equation (1) to Equation (3)).

(1) $${SBA}_{P}=\frac{f_{P}\left(b,X\right)}{f_{P}\left(b,X\right)+f_{B}\left(b,X\right)}{SBA}_{t}+\varepsilon_{P}$$

(2) $${SBA}_{B}=\frac{f_{B}\left(b,X\right)}{f_{P}\left(b,X\right)+f_{B}\left(b,X\right)}{SBA}_{t}+\varepsilon_{B}$$

(3) $${SBA}_{t}=f_{t}\left(b,X\right)+\varepsilon_{t}$$

where SBA_t_, SBA_P_, and SBA_B_ are the total SBA (m^2^·ha^−1^), SBA of *P. davidiana* (m^2^·ha^−1^), and SBA of *B. platyphylla* (m^2^·ha^−1^), respectively; X is the vector of independent variables; b is the parameter to be estimated; $f_{t}$, $f_{P}$ and $f_{B}$ are the basic models of SBA, *P. davidiana* SBA, and *B. platyphylla* SBA, respectively; $\varepsilon_{t}$, $\varepsilon_{P}$ and $\varepsilon_{B}$ are the error terms of SBA, *P. davidiana* SBA, and *B. platyphylla* SBA, respectively.

#### 4.3.3. Nonlinear Seemingly Unrelated Regression (NSUR)

Unlike AP, NSUR allows the effective additivity of SBA and considers the intrinsic correlation between the SBA values of multiple tree species [58]. The application of NSUR also reduces the confidence interval of parameter estimation values and improves the accuracy of model prediction [102]. Consequently, NSUR has been widely applied in biomass models [103,104] and crown width models [58]. The current study adopted NSUR (Equation (4)) to investigate the additivity of the SBA of *P. davidiana* × *B. platyphylla* broad-leaved mixed forests.

(4) $$\left\{\begin{array}{c} S{BA}_{P}=f_{P}\left(b,X\right)+\varepsilon_{P}\\ {SBA}_{B}=f_{B}\left(b,X\right)+\varepsilon_{B}\\ \;{SBA}_{t}={SBA}_{P}+{SBA}_{B}+\varepsilon_{t} \end{array}\right.$$

where SBA_t_, SBA_P_, and SBA_B_ are the total SBA (m^2^·ha^−1^), SBA of *P. davidiana* (m^2^·ha^−1^), and SBA of *B. platyphylla* (m^2^·ha^−1^), respectively; X is the vector of independent variables; b is the parameter to be estimated; $f_{P}$ and $f_{B}$ are the basic models of *P. davidiana* SBA and *B. platyphylla* SBA, respectively; $\varepsilon_{t}$, $\varepsilon_{P}$ and $\varepsilon_{B}$ are the error terms of totsl SBA, *P. davidiana* SBA, and *B. platyphylla* SBA, respectively.

#### 4.3.4. Model Fitting and Evaluation

The present study applied the Proc NLIN [105] and Proc MODEL [102] procedures in SAS 9.4 statistical analysis software to fit nonlinear regression models, AP models, and NSUR models of SBA. The accuracy of the model predictions was assessed via ten-fold cross-validation [92]. The goodness-of-fit of the SBA models was evaluated by the mean absolute error (MAE), mean percentage error (MPE), root mean square error (RMSE), coefficient of determination (R^2^), and adjusted coefficient of determination ($R_{adj}^{2}$).

(5) $$MAE=\frac{1}{n}\sum_{i=1}^{n}\left|y_{i}-{\hat{y}}_{i}\right|$$

(6) $$MPE=\frac{\sum_{i=1}^{n}|y_{i}-{\hat{y}}_{i}|}{\sum_{i=1}^{n}y_{i}}\times 100\%$$

(7) $$RMSE=\sqrt{\frac{{\sum_{i=1}^{n}\left(y_{i}-{\hat{y}}_{i}\right)}^{2}}{n}}$$

(8) $$R^{2}=1-\left[\frac{\Sigma_{i=1}^{n}{\left(y_{i}-\hat{y}\right)}^{2}}{\Sigma_{i=1}^{n}{\left(y_{i}-\bar{y}\right)}^{2}}\right]$$

(9) $$R_{adj}^{2}=1-\frac{n-1}{n-p}\left(1-R^{2}\right)$$

where $y_{i}$ is the observed SBA (m^2^·ha^−1^) of the i-th plot; $\bar{y}$ is the mean SBA (m^2^·ha^−1^); ${\hat{y}}_{i}$ is the predicted SBA (m^2^·ha^−1^); n is the number of samples; and p is the count of model parameters.

## 5. Conclusions

Based on this study, the Richards model (M_6_) and Korf model (M_1_) were shown to be optimal for predicting the SBA of *P. davidiana* and *B. platyphylla*, respectively, in complex structured *P. davidiana* × *B. platyphylla* broad-leaved mixed forests. The SBA prediction model was constructed based on AP and NSUR, incorporating SI, SDI, and ADBH. Compared to AP, NSUR not only resolved the additivity issue of SBA models for different tree species in mixed forests but also enhanced the prediction accuracy and universality of basal area models at both tree species and stand levels. The results of this study provide a scientific basis for accurately predicting SBA and optimizing stand structure.

## Figures and Tables

**Table 1:** A summary of the results of fitting theoretical models for predicting the stand basal area of *Populus davidiana* × *Betula platyphylla* broad-leaved mixed forests.

Species	Type	Model	Model Forms	b_0_	b_1_	b_2_	b_3_	b_4_	b_5_
SBA_P_	Korf	M_1_	$SBA=b_{0}{SI}^{b_{1}}exp\left(-b_{2}/ADBH\right){SDI}^{b_{3}}$	0.013 *** (0.002)	0.289 *** (0.032)	4.454 *** (1.042)	1.029 *** (0.020)	-	-
		M_2_	$SBA=b_{0}{SI}^{b_{1}}exp\left(-b_{2}/ADBH\right){\left(SDI/1000\right)}^{b_{3}}$	14.026 *** (1.661)	0.290 *** (0.034)	2.196 ** (0.769)	1.020 *** (0.020)	-	-
	Schumacher	M_3_	$SBA=exp\left(b_{0}+b_{1}/ADBH\right){SDI}^{b_{2}+b_{4}/ADBH}{SI}^{b_{3}+b_{5}/ADBH}$	−3.377 *** (0.650)	−30.547 ^ns^ (16.450)	0.801 *** (0.123)	0.452 ** (0.162)	6.049 ^ns^ (3.223)	−4.446 * (4.101)
		M_4_	$SBA=exp\left(b_{0}+b_{1}/ADBH\right){\left(SDI/1000\right)}^{b_{2}+b_{4}/ADBH}\;{SI}^{b_{3}+b_{5}/ADBH}$	2.105 *** (0.257)	12.677 * (6.345)	0.894 *** (0.093)	0.485 *** (0.086)	3.559 ^ns^ (2.385)	−5.384 * (2.224)
	Richards	M_5_	$SBA=b_{0}{SI}^{b_{1}}{\left[1-exp\left(-b_{2}{SDI}^{b_{3}}ADBH\right)\right]}^{b_{4}}$	31.156 * (13.690)	0.216 *** (0.043)	0.002 ^ns^ (0.004)	2.243 *** (0.271)	0.483 *** (0.066)	-
		M_6_	$SBA=b_{0}{SI}^{b_{1}}{\left\{1-exp\left[-b_{2}{\left(SDI/1000\right)}^{b_{3}}ADBH\right]\right\}}^{b_{4}}$	23.576 * (18.991)	0.288 *** (0.032)	0.001 * (0.001)	6.593 *** (1.808)	0.157 *** (0.041)	-
SBA_B_	Korf	M_1_	S$BA=b_{0}{SI}^{b_{1}}exp\left(-b_{2}/ADBH\right){SDI}^{b_{3}}$	0.012 *** (0.002)	0.285 *** (0.033)	3.867 ** (1.131)	1.030 *** (0.020)	-	-
		M_2_	$SBA=b_{0}{SI}^{b_{1}}exp\left(-b_{2}/ADBH\right){\left(SDI/1000\right)}^{b_{3}}$	10.301 *** (1.770)	0.423 *** (0.062)	1.443 ^ns^ (0.893)	1.034 *** (0.017)	-	-
	Schumacher	M_3_	$SBA=exp\left(b_{0}+b_{1}/ADBH\right){SDI}^{b_{2}+b_{4}/ADBH}{SI}^{b_{3}+b_{5}/ADBH}$	−4.862 *** (1.308)	1.667 ^ns^ (4.418)	1.073 *** (0.110)	0.374 ** (0.334)	−1.466 ^ns^ (3.450)	1.531 ^ns^ (8.214)
		M_4_	$SBA=exp\left(b_{0}+b_{1}/ADBH\right){\left(SDI/1000\right)}^{b_{2}+b_{4}/ADBH}\;{SI}^{b_{3}+b_{5}/ADBH}$	2.163 *** (0.414)	2.644 ^ns^ (9.992)	1.053 *** (0.074)	0.505 ** (0.183)	−0.648 ^ns^ (2.272)	−2.134 ^ns^ (4.570)
	Richards	M_5_	$SBA=b_{0}{SI}^{b_{1}}{\left[1-exp\left(-b_{2}{SDI}^{b_{3}}ADBH\right)\right]}^{b_{4}}$	16.121 ^ns^ (40.244)	0.775 ^ns^ (0.474)	0.013 ^ns^ (0.047)	1.014 ^ns^ (1.597)	0.424 ^ns^ (0.623)	-
		M_6_	$SBA=b_{0}{SI}^{b_{1}}{\left\{1-exp\left[-b_{2}{\left(SDI/1000\right)}^{b_{3}}ADBH\right]\right\}}^{b_{4}}$	19.954 ^ns^ (42.750)	0.728 ^ns^ (0.417)	0.002 ^ns^ (0.063)	0.537 ^ns^ (0.317)	0.931 ^ns^ (0.828)	-

Note: SBA is stand basal area (m^2^·ha^−1^); SBA_P_ and SBA_B_ are the SBA of *P. davidiana* (m^2^·ha^−1^) and SBA of *B. platyphylla* (m^2^·ha^−1^), respectively; SDI is stand density index (trees·ha^−1^) [64], SDI = N (D_0_/D_g_)^−1.605^; N is number of trees per hectare (trees·ha^−1^); D_0_ is standard base diameter (20 cm); D_g_ is quadratic average diameter at breast height (cm); SI is dominant height (m); ADBH is age at 1.3 m above ground level (year); b_0_–b_5_ are parameters of basal area model; ns means not significant; “***”, “**”, and “*” are significant at the levels of 0.001, 0.01, and 0.05, respectively; the standard error is in brackets.

**Table 2:** A summary of the results of evaluating theoretical models for predicting the stand basal area of *Populus davidiana* × *Betula platyphylla* broad-leaved mixed forests.

Species	Type	Model	MAE	MPE	RMSE	R^2^	$R_{adj}^{2}$
SBA_P_	Korf	M_1_	1.000	0.073	1.710	0.961	0.959
		M_2_	1.003	0.076	1.737	0.960	0.958
	Schumacher	M_3_	0.988	0.072	1.642	0.962	0.957
		M_4_	1.572	0.074	1.722	0.952	0.948
	Richards	M_5_	1.837	0.137	2.232	0.932	0.927
		M_6_	0.973	0.070	1.438	0.965	0.962
SBA_B_	Korf	M_1_	0.515	0.091	0.749	0.963	0.961
		M_2_	0.612	0.109	0.878	0.959	0.957
	Schumacher	M_3_	0.535	0.093	0.808	0.951	0.947
		M_4_	0.641	0.114	0.808	0.950	0.946
	Richards	M_5_	2.893	0.382	3.558	0.475	0.436
		M_6_	2.418	0.348	3.331	0.443	0.401

Note: SBA_P_ and SBA_B_ are the SBA of *P. davidiana* (m^2^·ha^−1^) and SBA of *B. platyphylla* (m^2^·ha^−1^), respectively.

**Table 3:** Parameter estimation of adjustment in proportion (AP) for predicting the stand basal area of *Populus davidiana* × *Betula platyphylla* broad-leaved mixed forests.

SBA	b_0_	b_1_	b_2_	b_3_	b_4_	RMSE	$R_{adj}^{2}$
SBA_P_	19.254 * (10.378)	0.307 *** (0.049)	0.003 * (0.001)	6.808 * (2.801)	0.157 * (0.065)	0.709	0.981
SBA_B_	0.010 * (0.004)	0.377 *** (0.081)	3.343 * (1.637)	1.032 *** (0.029)	-	0.678	0.979
SBA_t_	0.016 *** (0.005)	0.264 *** (0.048)	3.155 ** (1.201)	1.001 *** (0.028)	-	1.776	0.939

Note: SBA_t_, SBA_P_, and SBA_B_ are the total SBA (m^2^·ha^−1^), SBA of *P. davidiana* (m^2^·ha^−1^), and SBA of *B. platyphylla* (m^2^·ha^−1^), respectively; “***”, “**”, and “*” are significant at the levels of 0.001, 0.01, and 0.05, respectively; the standard error is in brackets.

**Table 4:** Parameter estimation of nonlinear seemly unrelated regression (NSUR) for predicting the stand basal area of *Populus davidiana* × *Betula platyphylla* broad-leaved mixed forests.

SBA	b_0_	b_1_	b_2_	b_3_	b_4_	RMSE	$R_{adj}^{2}$
SBA_P_	30.3645 * (13.4303)	0.2701 *** (0.0315)	0.0002 * (0.0001)	6.6979 *** (1.8965)	0.1498 *** (0.0429)	0.6297	0.9870
SBA_B_	0.0080 ** (0.0026)	0.4216 *** (0.0615)	1.4756 * (0.8851)	1.0338 *** (0.0210)	-	0.4092	0.9858
SBA_t_	-	-	-	-	-	0.6263	0.9757

Note: SBA_t_, SBA_P_, and SBA_B_ are the total SBA (m^2^·ha^−1^), SBA of *P. davidiana* (m^2^·ha^−1^), and SBA of *B. platyphylla* (m^2^·ha^−1^), respectively; “***”, “**”, and “*” are significant at the levels of 0.001, 0.01, and 0.05, respectively; the standard error is in brackets.

**Table 5:** Evaluation of the accuracies of models using different parameter estimation methods for predicting stand basal area.

Method	SBA	MAE	MPE	RMSE	R^2^	$R_{adj}^{2}$
NLS	SBA_P_	0.973	0.071	1.438	0.965	0.963
	SBA_B_	0.515	0.091	0.749	0.963	0.961
	SBA_t_	1.354	0.069	1.776	0.942	0.939
AP	SBA_P_	0.560	0.043	0.709	0.982	0.981
	SBA_B_	0.511	0.079	0.678	0.980	0.979
	SBA_t_	0.921	0.047	1.170	0.965	0.960
NSUR	SBA_P_	0.524	0.036	0.630	0.987	0.987
	SBA_B_	0.353	0.057	0.409	0.986	0.986
	SBA_t_	0.481	0.044	0.626	0.979	0.976

**Table 6:** Statistical summary of stand-level and tree species-level data for *Populus davidiana* × *Betula platyphylla* broad-leaved mixed forests.

Stand Variable	*P. davidiana* × *B. platyphylla*				*P. davidiana*				*B. platyphylla*			
	Max.	Min.	S.D.	Mean	Max.	Min.	S.D.	Mean	Max.	Min.	S.D.	Mean
SBA (m^2^·ha^−1^)	33.1	5.6	5.6	19.1	28.5	3.1	6.0	12.9	14.6	2.7	2.8	6.2
SI (m)	24.0	8.1	3.7	15.8	23.6	7.1	3.9	14.7	21.4	8.1	3.2	14.8
D_g_ (cm)	21.8	6.3	3.9	15.3	23.6	6.2	4.1	14.8	27.6	6.8	5.2	16.1
ADBH (year)	42	17	5	28	44	14	7	26	45	13	6	29
SDI (trees·ha^−1^)	985.8	317.4	245.7	698.3	849.8	105.8	185.8	469.2	547.1	50.6	183.1	242.7

Note: SBA is stand basal area (m^2^·ha^−1^); D_g_ is quadratic average diameter at breast height (cm); SI is dominant height (m); ADBH is age at 1.3 m above ground level (year); SDI is stand density index (trees·ha^−1^), SDI = N (D_0_/D_g_)^−1.605^; N is number of trees per hectare (trees·ha^−1^); D_0_ is standard base diameter (20 cm).

**Table 7:** Basic theoretical model for predicting the stand basal area of *Populus davidiana* × *Betula platyphylla* broad-leaved mixed forests.

Type	Expression	Reference
Korf	$SBA=b_{0}exp\left({-b_{1}t}^{{-b}_{2}}\right)$	Pan et al., 2023 [99]
Schumacher	$SBA=b_{0}exp\left(-b_{1}/t\right)$	Schumacher, 1939 [25]
Richards	$SBA=b_{0}{\left[1-exp\left(-b_{1}t\right)\right]}^{b_{2}}$	Richards, 1959 [100]

Note: SBA is stand basal area (m^2^·ha^−1^); b_0_, b_1_, and b_2_ are parameters of the model.

## Data Availability

Data are contained within the article.
